# Breast Cancer Resistance Mechanisms: Challenges to Immunotherapy

**DOI:** 10.1007/s10549-021-06337-x

**Published:** 2021-07-28

**Authors:** Ann Hanna, Justin M. Balko

**Affiliations:** Department of Medicine, Vanderbilt University Medical Center, Nashville, TN; Department of Medicine, Breast Cancer Research Program, Vanderbilt University Medical Center, Nashville, TN

**Keywords:** Immunotherapy, immune checkpoint blockade, resistance mechanisms, tumor microenvironment

## Abstract

The clinical implementation of immunotherapy has profoundly transformed cancer treatment. Targeting the immune system to mount anti-tumor responses can elicit a systemically durable response. Employing immune checkpoint blockade (ICB) has suppressed tumor growth and vastly improved patient overall and progression-free survival in several cancer types, most notably melanoma and non-small cell lung carcinoma. Despite widescale clinical success, ICB response is heterogeneously efficacious across tumor types. Many cancers, including breast cancer, are frequently refractory to ICB. Moreover, of initially ICB-responsive tumors, many acquire resistance due to tumor-specific alterations, loss of tumor-specific antigens, and extrinsic mechanisms that reshape the immune landscape within the tumor microenvironment (TME). The tumor-immune interaction circumvents the benefits of immunotherapy; tumors rewire the tumor-suppressive functions of activated immune cells within their stroma to propagate tumor growth and progression. In this review, we will discuss the challenges facing immunotherapy success and address the underlying mechanisms responsible for primary and acquired breast cancer resistance to immunotherapy.

## Introduction

The power of the immune system to combat tumor formation and progression has been extensively studied and debated over the last century. Historically, cancer patients with postsurgical infections had better survival outcomes, leading to the hypothesis that infection-associated activation of the immune system resulted in collateral anti-tumor effects [1]. Decades later, Thomas Lewis and Frank MacFarlane proposed that similar to host-vs-graft disease, cancer and immune cells interact as part of cancer immunosurveillance [2]. The concept of cancer immunosurveillance stated that an intact immune system will eliminate most nascently transformed cells, eventually selecting for resistant cell populations capable of polarizing normal immune system functions to permit and even foster tumor growth. Further studies identified three distinct phases associated with this phenomenon: elimination, equilibrium, and escape; collectively known as “cancer immunoediting” [3].

Years of research cemented the importance of the immune system in controlling cancer and prompted the exhaustive evaluation of different approaches to therapeutically mount patient anti-tumor immunity. The discovery and subsequent success of ICB was a pivotal milestone for cancer treatment; it elicited a robust systemic response, indiscriminate of tumor type, underlying etiology, and disease stage. However, the clinical response in patients has varied widely depending on tumor immunogenicity and the prevalence of lymphocyte infiltration into tumor stroma. ICB has largely increased patient survival in melanoma, non-small cell lung carcinoma, renal cell carcinoma, lymphoma, and head and neck squamous cell carcinoma [4]. Unlike most immunotherapy-responsive tumors, breast cancers harbor lower mutational burdens and generally exhibit limited lymphocyte infiltration, both of which suggest reduced immunogenicity and have led to the perception that breast tumors are characteristically “immune cold”. This generalization overlooks breast cancer heterogeneity, diversity of molecular subtypes, patient disease status, which step in the metastatic cascade tumors are detected, and complexity of the TME. To date, ICB is only indicated for advanced stage, metastatic triple negative breast cancer (TNBC), though activity was also observed in early stage TNBC. Despite promising advances in TNBC, immunotherapy response in breast cancer remains modest and unpredictable, particularly across molecular subtypes. ICB benefit in breast cancer was first demonstrated in the IMpassion130 trial, where adding atezolizumab (anti-PD-L1) to standard-of-care nab-paclitaxel increased progression-free survival in TNBC patients with ≥1% PD-L1+ immune cells in the tumor [5]. Subsequently, the KEYNOTE-355 trial employing pembrolizumab (anti-PD-1) in combination with different neoadjuvant chemotherapies (paclitaxel, nab-paclitaxel, or gemcitabine and carboplatin) enhanced progression-free survival in patients with untreated, inoperable, and metastatic TNBC with a combined positive score (CPS) of ≥10% PD-L1+ tumors [6].

Until recently, most breast tumors were traditionally considered immunologically quiescent. TNBC was the only subtype with prognostic TIL activity that translated to better patient outcomes [7], while largely being a negative biomarker for survival in hormone positive (HR+) tumors [8, 9]. However, the detailed characterization of immune signatures in a breast cancer patient cohort by Thorsson *et al* revealed that breast tumors range from inflamed and highly immunogenic (TNBC) to intermediate (HR+ breast cancer), therefore pointing to other confounding factors for poor immunotherapy response in addition to TIL infiltration alone. These discrepancies in tumor responses stem from the different resistance mechanisms that cancers deploy to either prevent sensitivity to immunotherapy (primary resistance) or circumvent an initial favorable response (acquired resistance). Immunotherapy resistance occurs through multiple modalities. Tumor cells may alter their proliferative capacity and suppress neoantigen formation, which impair immune recognition. In contrast, tumor cells can externally alter stromal and immune cells within the TME to promote tumorigenic, immunosuppressive functions.

## Overview of anti-tumor immunity

Anti-tumor immunity engages both arms of the immune system: innate and adaptive immunity. Per the cancer immunoediting theory, tumor cells dynamically interact with immune cells. In the early stages of tumorigenesis, an inflammatory response activates antigen presenting cell (APC) expansion to engulf and eliminate pathogens or aberrant cells through innate immunity.

The immune system can recognize mutated peptides expressed by aberrant cells as ‘non-self’. Human leukocyte antigen class I (HLA-I/MHC-I), comprised of HLA-A, -B, and -C, binds endogenous peptides via active ATP transporters (TAP1/2) that move pre-processed peptides from the (tumor cell) cytoplasm into the endoplasmic reticulum where they can be further processed and loaded onto MHC-I for trafficking to the cell surface. Peptide-MHC complexes (pMHC) can be bound by cognate T cell receptors (TCRs) complexed with CD8 or CD4 (for HLA-I and HLA-II respectively) to trigger anti-tumor immune function. This function includes direct cytotoxicity, mediated by CD8+ cytotoxic T lymphocytes (CTLs) or effector CD4+ T cell responses that supplement CD8 T cell cytotoxicity through supportive cytokine release. In addition to direct T cell recognition of tumor cells, APCs, including dendritic cells, B cells, and macrophages, can utilize exogeneous tumor antigens generated from turnover of proliferating or dying tumor cells and present them through human leukocyte antigen class II (HLA-II), or MHC-II (consisting of HLA-DR, -DQ, and -DP). Of all APCs, dendritic cells are also capable of cross-presenting (transporting externally-acquired peptides into the cytoplasm for proteasomal processing, as would be required for MHC-I-associated peptides) MHC-I antigens to CD8 T cells [10], which is particularly important for T cell priming in the tumor microenvironment or draining lymph node. In addition to MHC-TCR engagement (signal 1), costimulatory molecules CD80 and CD86, CD40, OX40, and ICOS expressed on APCs must bind to co-receptors CD28, CD40L, OX40L, and/or inducible T-cell costimulator ligand (ICOSL) (signal 2) on T cells to permit full T cell activation. Upon antigen presentation and costimulatory signaling, IL-12 activates a signaling cascade to promote T lymphocyte differentiation and expansion. Neoantigens due to genetic aberrations in tumor cells can also be presented on HLA-II to helper T cells, which can secrete cytokines to functionally activate members of the innate immune system, namely natural killer (NK) cells, dendritic cells (DCs), and macrophages, to induce apoptosis in tumor cells. Due to intratumor heterogeneity, tumor cell variants may survive the initial anti-tumor immune response and remain quiescent, reaching equilibrium under the selective pressure of anti-tumor immune surveillance.

During equilibrium, dormant tumor cells develop intrinsic modifications that promote cellular division and immune evasion. These mutations include overexpressing proliferation genes, downregulating antigen expression, secreting immunosuppressive cytokines, and upregulating immune checkpoint ligands; all of which impede overall immunity. These mechanisms allow tumors to create favorable secondary niches for the metastasis of disseminated tumor cells. Such advantages facilitate the eventual progression of cancer cells from quiescently controlled, to clinically detectable tumors. Unrestrained tumors can also secrete cytokines to rewire the immunogenic functions of immune cells to establish an immunosuppressive local microenvironment for escape and ultimate progression.

## Tumor-specific resistance

### Tumor antigenicity and mutational burden

A fundamental tenet of adaptive immunity necessitates the recognition of foreign or aberrant cellular antigens that activate APCs to process and present antigens to T lymphocytes. Generally, genomic instability and accumulation of somatic mutations within DNA coding regions are hallmark features of malignant cells. These genetic anomalies can cause minor alternations, like single base substitutions, or major structural rearrangements, fusions, insertions, deletions, and variants. Antigen peptide sequences distinguishing tumor cells are categorized based on unique cell expression patterns. Tumor-specific antigens (TSA) are restricted to tumor cells compared to their non-transformed counterparts (*e.g*. neoantigens formed by mutations). Genomic aberrations often target genes that drive functional tumorigenic advantages. For example, the loss of function silencing of tumor-suppressor genes, like *ATM, PTEN, P53, LKB1*, and *CHEK2*, allows tumor cells to bypass cell-cycle checkpoint inhibitors to promote cellular proliferation. Furthermore, breast tumorigenesis is often associated with mutations in hallmark DNA repair genes like NBS-1, BRCA1 and BRCA2. Each of these processes, both ‘driver’ mutations benefitting the tumor, and ‘passenger mutations’ – those resulting from acquired genomic instability with no known tumorigenic function – can lead to TSAs. In contrast, tumor-associated antigens (TAA) are not unique to but are instead overexpressed on tumor cells. Human epidermal growth factor-receptor 2 (HER2)-amplification is a major breast cancer TAA for generating an immune response. Additionally, hallmark immunogenic breast cancer TAAs fall into many categories. Overexpressed oncofetal antigens (e.g., CEA) are proteins found in fetal tissues during development and are typically silenced in adult, however, are expressed in malignant somatic cells. Post-translationally modified antigens are overexpressed surface marker proteins, such as glycolipids like MUC-1. Non-somatic antigens (e.g., MAGE) are germ cell-specific genes that may be expressed on malignant cells. Differentiation antigens (e.g., NY-BR-1 and WT1) are proteins associated with terminal differentiation of tissue-specific cells that are found on tumor cells and non-transformed cells of the same lineage [11].

Antigen presentation and recognition via a cognate TCR prompts the clonal expansion and activation of tumor antigen-specific T cells which can efficiently impair tumor growth. Thus, tumor neoantigen formation, release, and processing are indispensable for eliciting an anti-tumor immune response. Tumors can shed various peptides, through various processes like exosome secretion or via cell turnover (e.g. cell death and necrosis) which are subsequently phagocytosed by APCs. However, only properly processed peptides, such as those cleaved to the correct length and structure, are subsequently loaded onto MHC molecules. Thus, the mutational load can only be beneficial for driving an immune response if: 1) tumor cells shed high numbers of neoantigens, 2) neoantigens are phagocytosed by APCs, 3) antigen peptides are processed and loaded onto MHC molecules on the surface of APCs, 4) APCs successfully engage T cells through MHC-TCR interactions, and finally, 5) the presence of co-stimulatory signaling.

Although tumors usually have high mutation burdens, they can alter every step of the antigen generation and processing pathways to evade immune surveillance [12]. Tumor cells often downregulate, mutate [13], or completely lose immunogenic antigens [14, 15] through suppressing antigen processing machinery that mediate antigen transport and cleavage [16]. Breast cancers downregulate TAP1 [17], TAP2 [18], and TAPBP [19] expression; all of which are critical transporters necessary for antigen transport across the ER to be loaded onto MHC molecules. Breast tumors harbor loss of heterozygosity and epigenetic silencing of some MHC-I molecules [20] effectively suppressing antigen recognition and presentation to T lymphocytes. Disruption of this pathway is often associated with poor clinical outcomes [21]. The epigenetic suppression of MHC-I molecule expression can be clinically targeted through the use of DNA methyltransferases to restore expression and potentially anti-tumor immunity[22]. Interestingly, tumors rarely lose MHC I expression altogether to escape immunity, since the lack of MHC-I indicates an inherent “missing self” signal, compelling NK cells to eliminate tumor cells. Moreover, tumors can change the expression of MHC subunits, such as beta-2-microglobulin (B2M), causing MHC misfolding and inability to bind antigens [23].

### Aberrant signaling pathways

The mutational properties and epigenetic abnormalities that confer proliferative and invasive capacities have been extensively studied in breast cancer. However, tumors can also modulate key signaling pathways to alter antigen presentation, attenuate an immunogenic inflammatory response, and inhibit lymphocyte recruitment into tumors to evade immunologic detection and cytotoxicity. In addition, tumors can dysregulate signaling pathways to suppress apoptosis, consequently promoting survival and downregulating neoantigen release.

Interferon γ (IFN-γ) signaling is critical for normal immunity against pathogens and cancer [24, 25]; its deficiency results in severe microbial and viral susceptibilities [26]. NK, effector T cells, and APCs produce IFN-γ to activate inflammatory pathways via signal transducers and activators of transcription-1 (STAT1) signaling. STAT1 promotes the transcription of IFN target genes like TNF-α, iNOS, COX-2 and IL-1β; all of which enhance antigen presentation and MHC expression [27]. Additionally, IFN-γ can directly induce apoptosis or cytostatic behavior in tumor cells [28], therefore, tumors often downregulate or mutate proteins involved in the IFN-γ signaling cascade, including IFN-γ receptors, STAT1, and JAK1/2 [29–31]. To control exacerbated immune responses, IFN-γ activation transcriptionally regulates PD-L1 expression as negative feedback mechanism; moreover, mutations in the IFN-γ pathway nullifies response to ICB [32]. As a result, prolonged IFN-γ exposure can lead to immune evasion by applying selective pressure [33].

Wnt/ β-catenin is another immunoregulatory pathway in cancer composed of a complex family of proteins that transduce signaling through ligand-dependent and -independent mechanisms [34]. Wnt activation culminates in the accumulation of transcriptional coactivator β-catenin to initiate the transcription of target genes including many cell cycle genes and oncogenes such as MYC. Breast cancer Wnt aberrations occur through the overexpression of pathway components: FZD, LRP 5/6, DDX, and ROR 1/2 [35, 36]; all of which can canonically and noncanonically activate Wnt. Dysregulated Wnt signaling drives invasiveness, epithelial-mesenchymal transition (EMT), pluripotency, enhancing motility, and providing cues for tumor cell proliferation [37]. Wnt can specifically attenuate anti-tumor immunity by decreasing the expression of CCL4, which is a potent chemoattractant, thus preventing the recruitment of DCs and T cells to the TME and blocking adaptive anti-tumor immunity [38]. In TNBC, Wnt signaling correlates with a “stemness” phenotype and increased PD-L1 expression [39]. Consistent with these findings, tumors with elevated Wnt signaling often display blunted responses to ICB.

The mitogen-activated protein kinase (MAPK) pathway is commonly dysregulated in several tumor types, conferring oncogenic and proliferative advantages upon growth factor-cell surface receptor engagement [40]. MAPK activation (e.g. via K-Ras and B-Raf) upregulates VEGF, IL-18, and CXCL1/2, which promote tumorigenesis, angiogenesis, inhibit TIL recruitment, and mediate MDSC recruitment [41–43]. MAPK inhibition can upregulate MHC-I, MHC-II, and PD-L1 expression under basal and IFN-γ - stimulated conditions, depending on context, and can enhance TIL infiltration [44]. Additionally, MEK inhibitors have been reported to activate STAT1 signaling in mammary tumor cell line, inducing tumor immunogenicity through MHC-I and PD-L1 expression. [45]. Combining MEK inhibitors with ICB synergistically enhances the ICB-induced anti-tumor response in preclinical models [46].

The oncogenic activation of various signaling pathways is implicated in tumor progression and breast cancer therapeutic resistance. Dysregulated Notch signaling sustains TNBC growth by promoting angiogenesis, upregulating cell cycle genes that trigger cell division, and inducing stem cell maintenance [47]. Preliminary studies point to the immunological benefit of paracrine Notch activation in macrophages, as it permits macrophage-mediated phagocytosis [48]. Oncogenic Hippo inactivation promotes proliferation, invasion, and EMT in breast cancer [49]. Hippo signaling also drives breast cancer metastasis by regulating a hypoxic microenvironment in bones, inducing therapeutic resistance, and modulating immune evasion by upregulating PD-L1 in tumor cells [50]. Hedgehog signaling orchestrates M2 macrophage polarization to create an immunosuppressive TME [51], through blocking CTL recruitment [52], thus allowing tumor progression. Overall, the autocrine and paracrine activation of developmental signaling may contribute to the immunological escape of quiescent tumor cells and recurrence post-therapy due to proliferative advantages and the establishment of an immunosuppressive systemic response.

The ability of immunogenic pathways to promote immune suppression reinforces the multifaceted functions of oncogenic signaling that drive therapeutic resistance. For example, the PD-L1 expression may be inherently upregulated to mutations or induced in response to aberrant oncogenic signaling. Constitutive PD-L1 upregulation on tumor cells is a result of epigenetic and posttranslational modifications. Many solid tumors, including breast cancer, harbor chromosomal amplifications (9p24.1) of amplicons containing PD-L1 ligands [53] and structural translocation of class II transactivator (CIITA), which encodes MHC expression, to the PD-L1 gene leading to upregulatedPD-L1 expression [54]. In breast cancer, differential methylation patterns in multiple immune checkpoint ligand promoters, including PD-L1, CTLA-4, LAG-3, and TIM-3 upregulate their expression compared to normal tissue and correlate with poor patient prognosis [55].

Inducible PD-L1 expression, however, results from pro-inflammatory signaling pathways, such as IFN-γ, TNF-α, and IL-6, that activate immunity and promote T cell effector and cytotoxic functions. Inflammation upregulates negative checkpoint inhibitor protein expression on immune cells (e.g. PD-1 on T cells and CD47 “do not eat me signal” on macrophages) as a negative feedback regulator to control exacerbated immune responses. This mechanism is co-opted by tumor cells within the tumor microenvironment to drive adaptive resistance in tumors. While PD-L1 expression may serve as a biomarker for patient selection to receive immunotherapy, it does not necessarily predict favorable patient response to anti-PD-1/anti-PD-L1 immunotherapy. Other mutations drive PD-L1 expression in solid tumors to inhibit immune cell activation, including MYC amplifications, PTEN deletions, and EGFR and AKT mutations [56]. Targeting these oncogenic pathways therapeutically may create an avenue for combinatorial approaches with immunotherapy to sensitize tumors with adaptive resistance to respond to treatment.

## Tumor microenvironment resistance

The composition of the TME, comprised of various cell types that foster diverse tumorigenic functions, plays a key role in influencing therapeutic response. As mentioned earlier, there is ongoing crosstalk between tumor and stromal cells. Tumors attenuate the production and secretion of various chemokines to block the recruitment and infiltration of TILs. If immune cell infiltration occurs, tumors can create an immunosuppressive local microenvironment permitting progression and metastasis [57, 58]. However, the abundance of immunosuppressive immune cell populations is not a reliable indicator for poor patient outcomes in response to therapy. More likely, the delicate balance between immunosuppressive and activated effector and cytotoxic T cells dictates response to therapy and the overall immunogenic status of the TME.

### Innate immune cells

Cells of the innate immune system perform necessary immunogenic functions to eliminate pathogens and foreign material. Innate immune cells include APCs-DCs and macrophages, NK cells, and neutrophils. DCs and macrophages express scavenger receptors that recognize endogenous and pathogenic proteins and consequently promote the phagocytosis and clearance of apoptotic cells and pathogens to maintain homoeostasis. Mature DCs and M1 macrophages express high levels of MHC-II and costimulatory ligands CD80, CD86 to ensure successful antigen capture and presentation to T cells. This process prompts the release of pro-inflammatory cytokines, including IL-6, IL-12, IFN-γ and TNF-α, to create a delayed type hypersensitivity-like microenvironment that regulates prolonged anti-tumor adaptive responses [59]. In pathogenic states, immature DCs and M2-polarized macrophages express low levels of MHC-II and costimulatory molecules, exhibit weak endocytosis, and lack the secretion of pro-inflammatory cytokines, downregulating immunity to maintain a wound-healing, reparative environment. Additionally, they express inhibitory checkpoint markers PD-L1 and CTLA-4 to inactivate T cells and promote anergy. Consequently, inactivated DCs and macrophages fail to process antigens and may be hijacked by tumors to suppress anti-tumor immunity, thus allowing tumor escape. Tumors can polarize macrophages toward an immunosuppressive M2 phenotype, better known as tumor-associated macrophages (TAMs). TAMs are abundantly found within the TME of many primary tumors, correlating with poor clinical prognosis and reduced survival. Normally, macrophages mount anti-pathogenic immune responses by initiating inflammation, however, prolonged inflammation is diverted by tumors to mediate reparative, immunosuppressive functions. Macrophages are co-opted by tumors to perform pro-tumorigenic functions through secretion of growth factors that promote tumor cell proliferation and differentiation. TAMs maintain tumor growth, angiogenesis, invasion, and migration by producing PDGF, VEGF, EGF, COX-2, and matrix metalloproteases (MMP) 2 and 9. In addition, TAMs secrete immunosuppressive cytokines and express immune checkpoint inhibitors, such as PD-L1, leading to T cell inactivation. TAMs competitively deplete T cell metabolic nutrients through upregulation of metabolic enzymes, Arg1, IDO1, and IDO2, which promotes T cell anergy [60, 61].

In response to pathogens, NK cells produce IFN-γ and TNF-α to activate macrophages and neutrophils to phagocytize foreign pathogens. NK cells produce perforin, granzymes, and other apoptosis-inducing proteins death ligands like tumor necrosis factor-related apoptosis inducing ligand (TRAIL) and Fas ligand (FasL), and thus can mediate cytotoxicity like CTLs. Since NK cells lack TCRs, they do not require antigen presentation for activation, however, the absence of MHC-I on normal cell surfaces, which provides a protective “self-signal”, activates NK cytolysis (termed a ‘missing self’ signal). NK cell abundance in cancer patients correlates with APC, CTL, and effector T cell infiltration and activation [62]. Despite the positive prognostic impact of NK cells, they can mediate regulatory functions by inducing angiogenesis and upregulating PD-L1 and LAG-3 to block T cell activation thus facilitating tumor escape.

Neutrophils are the first responders during inflammation to phagocytize and eliminate pathogens [63]. In response to IFN-β signaling, neutrophils secrete pro-inflammatory cytokines TNF-α and nitric oxide (NO) to induce anti-tumor cytotoxicity. Additionally, they activate T cells by overexpression CD86 and CD54 [64]. The potent inflammatory response neutrophils initiate is a major contributor to tumorigenesis. Upon encountering pathogens, neutrophil phagolysosomes produce NADPH, free radicals, and reactive oxygen species, all of which may induce genotoxic insults that damage DNA. Neutrophils also promote tumor invasion and angiogenesis through production of MMP9, elastase, VEGFA and oncostatin M [65]. Neutrophils can also suppress anti-tumor immunity by producing Arg1, a well-established suppressor of T cell function [66]. Enrichment of tumor associated neutrophils (TANs) within patient tumors is a negative prognostic marker. PD-L1-expressing neutrophils, common in TANs, can promote cancer metastasis [67]. TANs can form neutrophil extracellular traps (NETs), which are structures composed of decondensed chromatin and proteolytic enzymes that normally entrap and neutralize pathogens. NETs promote tumor cell adhesion, dissemination, and metastasis [68]. NET formation, termed NETosis, is found in both primary tumors and metastatic sites as NET components are overexpressed in various tumors and correlates with poor prognosis in breast carcinoma patients [69].

Immature myeloid cells give rise to a heterogeneous population of myeloid-derived suppressor cells (MDSCs) that are activated in a range of pathologic conditions and cancer. During acute infections, myeloid precursors differentiate into neutrophils and macrophages so they can activate phagocytosis. To resolve infections, MDSCs secrete immunosuppressive cytokines to initiate wound healing mechanisms; a process co-opted in cancer to induce proliferative signaling [70]. MDSC enrichment correlates with poor survival in different tumor types including pancreatic, breast, and lung carcinomas [71]. MDSC recruitment to the TME blocks cytotoxic and effector T cells and activates Treg recruitment through the production of anti-inflammatory cytokines IL-10, TGF-β, Arg1, and prostaglandin E2. Moreover, MDSCs suppress NK cells and DCs through the production of reactive oxygen species. In cancer, monocytic MDSCs can give rise to TAMs, while granulocytic MDSCs can give rise to TANs, thus replenishing the pool of immunosuppressive pro-tumorigenic cells [72].

Given the importance of innate immune cells in modulating tumor immunity, the paradoxical effects of inflammation are of note. In response to tumor-independent stressors such as pathogenic infections, environmental toxic exposure, obesity, and autoimmune disease, an inflammatory signaling cascade is initiated to activate innate and humoral immunity to restore homeostasis. Insults to epithelial cells activate myeloid cells to secrete inflammatory cytokines to promote epithelial cell proliferation and stem cell de-differentiation. Ideally, post injury or infection, a ‘wound-healing’ process is initiated. However, chronic exposure to inflammatory factors triggers cellular malignancy and progression by promoting tumor cell proliferation, survival, and angiogenesis. Tumor cells can self-sustain this supportive environment by producing inflammatory cytokines like TNF-α, IL-1β, and IL-6. Conversely, therapeutic inflammation can also be mediated by innate immune cells, e.g., NK cells and M1 macrophages, promoting anti-tumor immunosurveillance. Ultimately, however, sustained tumor microenvironment inflammation results in tumor cell proliferation and activation of immunoregulatory cells like MDSCs and Tregs, which dampen effector function. Thus, the plasticity of immune cells in the tumor microenvironment and duality of immunogenic functions must be delicately balanced in order to maintain homeostasis and activate anti-tumor immunosurveillance.

### Adaptive immune cells

T cell responses are determined by the stimulus they receive and the status of costimulatory signaling, allowing them the flexibility to perform cytotoxic, helper, or regulatory functions [73]. Specialized T cell subsets fight against pathogenic infections and cancer, while others maintain homeostasis and self-tolerance. T lymphocyte plasticity is particularly important for tumor progression. While the infiltration of T cells is a major prognostic and determinant of anti-tumor activity, tumors skew CTL functions, favoring immunosuppressive, regulatory activities to permit tumor immunoediting.

IFN-γ/STAT1 signaling activates effector type 1 helper T cells to promote immunity against biological pathogens including viruses, bacteria, or immunogenic cells. Further Th1 cell expansion occurs in response to IL-2/STAT4 signaling and antigen presentation. Th1 cells secrete pro-inflammatory cytokines that mediate a feedforward loop of Th1 activation and induce APCs. Consequently, APC activation allows them to perform phagocytic and APC functions and upregulates their MHC-I expression, thus directly priming CTL-mediated anti-tumor immunity. Th1 cells can also activate the complement system through impacting B cells. Cumulatively, Th1 cell-mediated functions are critical for inducing robust anti-tumor immunity [74].

Type 2 helper T cells are activated in allergic responses, helminthic infections, and wound healing responses in response to IL-4/STAT6-GATA3 signaling [75]. Th2 cells produce anti-inflammatory cytokines that stimulate humoral immunity through IgE secretion, thus activating eosinophils to release histamines and suppress inflammation. More specifically, Th2-derived IL-4, IL-10 and IL-13 block Th1 activation and expansion and polarize the transcriptional programs of TAMs and MDSCs to assume immunosuppressives phenotypes [76]. Th2 cell abundance within the TME correlates with a worse prognosis in patients with many cancer types.

Regulatory T cells (Tregs) are the main inhibitors of autoimmune, inflammatory, and graft-vs-host diseases [77]. Intra-tumor prevalence of Tregs correlates with poor prognosis and survival in many cancer patients [78]. Tregs mediate immune suppression through several potent mechanisms. Tregs secrete immunosuppressive cytokines TGF-β, IL-10, and IL-35 to mediate an anti-inflammatory state. Metabolically, they outcompete effector T cells for a limited pool of metabolites and deprive them from necessary nutrients. Moreover, Tregs directly induce apoptosis in effector and CTLs by secreting granzyme A and B, and perforin [79].

Other CD4 T cell subsets include Th17 and follicular helper T (Tfh) cells. Th17 cells secrete high levels of IL-17 [80] and other cytokines like CCL2, CCL20 IL-1*β*, and IL-6 [81–83] that mediate pro-inflammatory and regulatory functions [84]. The enrichment of Th17 cells in cancer benefits tumor growth by inducing angiogenesis via VEGF production or transdifferentiating into immunomodulatory Th2 or Tregs [85]. Tfh T cells regulate B cell antigen education in secondary lymphoid organs. Tfh produce pro-inflammatory cytokines IL-2 and IFN-γ to activate immunity. In cancer, Tfh cells associate with enhanced patient survival, increased CTL infiltration, and controlled tumor growth [86]. TNBC models treated with ICB display Tfh cell enrichment, which activates B cell-mediated anti-tumor immunity [87].

CD8+ T cells are the chief mediators of T cell-mediated cytotoxicity [88]; they differentiate and expand in response to IL-12/mTOR signaling. The initial activation of CD8 cells primarily results from antigen presentation and costimulatory signaling by DCs in lymph nodes. Upon antigen education, CD8 cells are activated by binding MHC I on tumor cells. The TCR-MHC interaction activates CTLs to release lytic granules consisting of cytotoxic proteins: perforin, granzymes A and B, and granulysin. Perforin attaches to and creates pores in the plasma membranes of target cells. Then, apoptosis-inducing serine proteases, granzyme proteins, enter target cells and cleave intracellular proteins to induce an apoptotic signaling cascade. Activated CD8 CTLs express TRAIL and FasL that can also directly activate apoptosis in target cells.

While the initial presentation of neoantigens by DCs to T cells occurs in the lymph node, the continuous exposure to inflammatory signals induces the development of peripheral tertiary lymphoid structures (TLS) in secondary tissues for fast and efficient immunogenic responses. TLS mediate response to many pathologies including chronic inflammatory syndromes and cancer. TLSs harbor endothelial cells, DCs, T and B cells, which compartmentalize similarly to germinal centers in the lymph nodes. In cancer, TLSs are adjacently located to tumors and can be composed of many cells that perform various immune functions depending on the tumor type and immune cell content [89]. TLS, in melanoma, NSCLC, and TNBC, are enriched for DCs and correlate with enhanced CTL infiltration, which activates antitumor response and favorable patient outcome. TLS in hepatocellular carcinoma, however, correlated with more aggressive, advanced disease. More recently, TLS harboring B cells have been discovered to co-occur with T cells in melanoma, where they determine their cytotoxic phenotype and correspond with favorable patient response to immunotherapy [90, 91]. The analysis of TLS in melanoma patients with complete response to ICB displayed patterns of clonal B cell expansion that determines long-term memory anti-tumor responses [92]. Furthermore, regardless of CTL infiltration in soft-tissue sarcoma, B cell-containing TLS sufficiently predicted favorable patient response to anti-PD-1 blockade [93]. These data support the emerging role for TLS and their composition as a prognostic and predictive biomarker for ICB response in cancer patients.

### Stromal Cells

In addition to lymphocyte infiltration, the breast tumor microenvironment is enriched for many stromal cells. The complex architecture of the breast, characterized by endogenous and recruited stromal cells, encompassing endothelial cells, mesenchymal stem cells, and cancer-associated fibroblasts (CAFs), significantly impacts tumor growth and progression. Stromal components engage in crosstalk with tumors via secreted soluble growth factors and cytokines that mediate various functions, such as angiogenesis, extracellular matrix remodeling, cellular extravasation and migration, and immune evasion, all of which enable metastasis.

Mesenchymal stroma cells (MSCs) are renewable pluripotent stromal calls that can differentiate into many types of cells like osteocytes, adipocytes, and chondrocytes. In breast cancer, MSCs secrete VEGF, SDF-1, and CCL5 [94], which enhance breast cancer cell mobility and invasiveness [95] and induce EMT in primary tumor cells, which aids in metastasis to secondary locations [96]. In response to TGF-β signaling in breast cancer, MSCs differentiate into CAFs [97]. The autocrine CAFs production of TGF-β induces EMT and breast cancer metastasis. In the tumor microenvironment, CAFs produce DDR2, a collagen receptor, that mechanically reshapes extracellular matrix stiffness to facilitate tumor cells migration and metastasis [98]. Analysis of mammary tumor specimens from early to late stage disease revealed dynamic longitudinal changes in CAF transcriptomic phenotypes that mediate therapeutic resistance and correlate to poor patient survival [99, 100].

## Considerations for variable immunotherapy responses

### Patient selection

A major reason for modest immunotherapy success in breast cancer is failure to preselect patients most likely to benefit from treatment. Initial trials evaluating combinatorial immunotherapy in a metastatic breast cancer setting demonstrated a survival benefit exclusively in patients whose tumor biopsies were positive for PD-L1 expression. Currently, the immunohistochemical verification of PD-L1 expression within the TME is the only criteria for immunotherapy eligibility (in the metastatic setting) and despite the PD-L1+ status of some patients, many do not demonstrate benefit to ICB. Thus, identifying novel biomarkers to more reliably predict patient subsets who will benefit from ICB is critical. Moreover, the discrepancy of PD-L1+ expression and favorable ICB response in patients suggests compensatory mechanisms breast tumors employ for immunotherapy resistance. Indeed, other inhibitory immune checkpoint ligands, like those of the B7-family (B7-H3 and B7-H4) can be upregulated in some patients’ tumors [101, 102].

Breast cancer patient candidates for immunotherapy (i.e., those in the first-line metastatic TNBC setting) have often progressed through chemotherapy (e.g. neoadjuvant chemotherapy [NAC]) or radiation treatment to treat the primary tumor. The neoadjuvant and post-surgical curative chemotherapy TNBC patients receive may significantly alter the immunologic landscape and adversely impact immunotherapy response in the metastatic setting. Ideally, chemotherapy and ionizing radiation induce immunogenic tumor cell death resulting in neoantigen release and detection by APCs, both of which trigger tumor-specific immune infiltration and activation [103]. NAC may enrich the infiltration of myeloid populations, which correspond to worse progression-free survival in breast cancer patients [104]. Detailed transcriptomic profiling of pre- and post-NAC treated breast tumors revealed significant, subtype-specific immune microenvironment changes [105]. TNBC patients displayed a higher immunogenic phenotype and augmented TIL abundance that correlated with pathologic complete response compared to HR+ breast cancer patients. Additionally, residual tumors were enriched for immunosuppressive cell populations like M2 macrophages and harbored less Th1 and CD8+ TILs. This evidence points to chemotherapy-induced immunoediting of the local TME, which could explain the mitigated response to second-line therapies like ICB. Therefore, ascertaining the most efficacious, immunogenic treatment combinations and deploying them earlier in therapy is likely essential for maximizing the therapeutic benefit for breast cancer patients. However, such approaches must weigh the risks of financial and physiological toxicity of ICB, and further reinforce efforts for specific biomarkers of ICB benefit.

### Insights from clinical trials

Despite the number of clinical trials evaluating the impact of immunotherapy in breast cancer, detailed correlative and molecular analyses of tumor specimens have not been reported. The reporting of larger Phase II and III breast cancer clinical trials have largely focused on ascertaining the safety and clinical outcomes in response to ICB, with little detailed tissue analysis beyond the presence of TILs or expression of PD-L1, which has enriched for responding patients in the metastatic setting but not the early setting. Overall, the modest response in breast cancer patients to monotherapy ICB indicates inherent tumor resistance. The molecular characterization of tumors pre- and post-treatment therapy may help elucidate mechanisms of adaptive tumor resistance to mono and combination therapy ICB.

Despite the limited information in breast cancer response to immunotherapy due to the relative short length of time ICB has been administered clinically in breast cancer patients, many conclusions can be drawn from clinical trials in other cancer types to explain acquired resistance to immunotherapy. The upregulation of checkpoint inhibitors LAG-3, TIM-3, TIGIT, and VISTA on T cells post anti-PD-L1 ICB has been described in many tumors, including melanoma and non-small cell lung carcinoma (NSCLC), as a mechanism for acquired resistance [106]. Furthermore, the employment of T cell based therapies induces loss-of-function mutations in critical signaling pathways that promote antigen presentation and T cell function, such as JAK1/2 and STAT1 mutations, to evade IFN-γ stimulation and impair T cell function in melanoma patients post anti-PD-L1 and anti-CTLA-4 [107]. In addition to immune-specific adaptive resistance, many tumors downregulate antigen presentation machinery in response to immunotherapy. Melanoma patients downregulate β2M, prompting MHC class I loss in response to PD-1 blockade, thus blocking tumor cell recognition by T cells. With more widescale implementation and evaluation of ICB in breast cancer, additional modalities of intrinsic and adaptive resistance mechanisms may be identified.

### Unexpected outcomes to immunotherapy

In addition to immune-related adverse effects (irAEs), or organ-specific toxicities, emerging adverse outcomes include the rapid acceleration of tumor growth rate. ICB causes unpredictable and opposing effects in certain patient subsets of different tumor types, whose tumor burden rapidly progresses, a phenomenon fittingly called “hyperprogressive disease” (HPD) [108]. Several studies implicated the mutagenic overexpression of MDM2, EGFR, and FGF as the underlying causes for HPD [109]. HPD was evaluated in a limited retrospective study of a small TNBC patient cohort, however, yielded no particular adverse survival or prognosis [110]. Clinically expanding the application of ICB in breast cancer patients would conceivably demonstrate similar, adverse outcomes.

## Conclusions

Although responses to immunotherapy can often be durable and robust, cancers inevitably develop diverse resistance mechanisms through evolving structurally or skewing the functions of virtually all cells of the immune system to promote progression. Resistance mechanisms, like reducing mutational load (i.e immunoediting) and neoantigen formation and presentation, form the first line of defense against the engagement of host immunity. If the initial layer of protection fails and tumors are detected by the immune system, tumors reshape their microenvironment through secreting cytokines or upregulating the expression of proteins to maintain an immunosuppressive state.

The implementation of ICB has drastically improved patient response and clinical outcome in NSCLC, melanoma, and renal cell carcinoma patients. The recent approvals of ICB in TNBC breast cancer have significantly enhanced patient outcomes and opened the door for extension to other molecular subtypes. Many clinical trials are underway to evaluate the efficacy of therapeutic combinations with ICB to deepen and broaden patient responses. Despite initial favorable responses, tumors acquire resistance mechanisms which converge to escape anti-tumor immunity incurred by immunotherapy. Thus, elucidating the molecular mechanisms that drive molecular and immunologic resistance and exploring potential combinatorial approaches are vital to enhance patient responses to therapy in the future.

## Figures and Tables

**Fig. 1:**
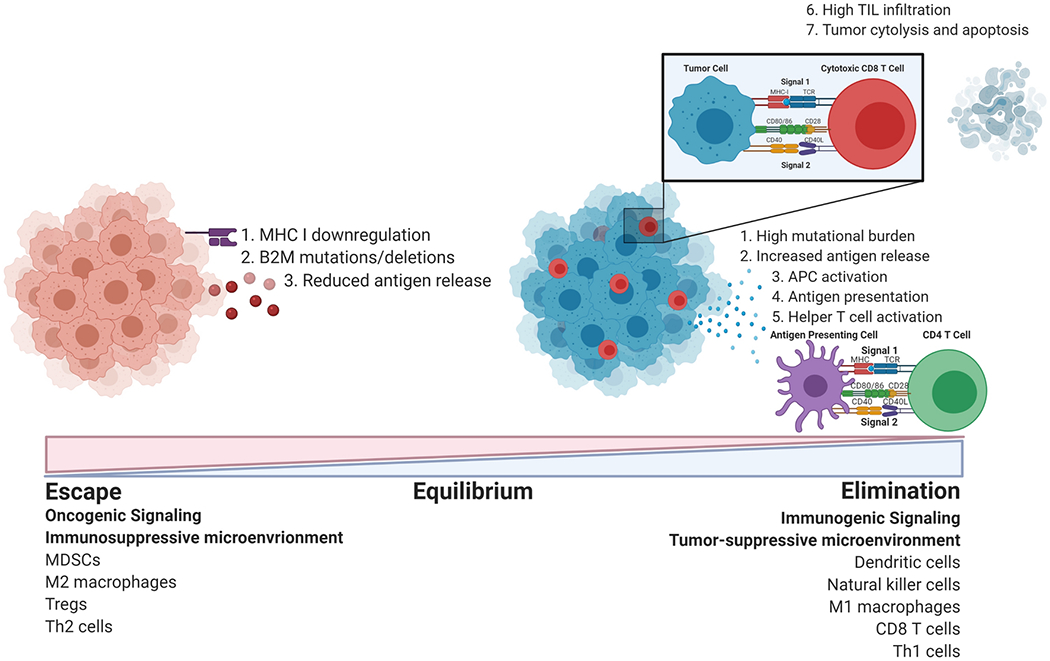
Anti-tumor immunity is initiated upon tumor cell death that releases tumor antigens for capture and processing by antigen presenting cells (dendritic cells, macrophages, B cells) for presentation to T cells through binding TCR (signal 1). Complete T cell activation requires the secondary engagement of costimulatory receptors (signal 2), which may also function as inhibitory checkpoints to suppress T cell activation. Successful antigen presentation allows tumor antigen-specific T cells to home to the primary tumor to perform cytolytic and effector functions to eliminate tumor cells. The escape of subpopulations of tumor cells may occur through aberrant signaling that alters neoantigen generation and release and impairs antigen processing machinery. Tumor-derived cues polarize immune populations (MDSCs, M2 macrophages, Tregs, Th2 cells) to perform pro-tumorigenic functions, leading to an anti-inflammatory, immunosuppressive state permissive of cancer progression.
